# Brucellosis in wildlife in Africa: a systematic review and meta-analysis

**DOI:** 10.1038/s41598-021-85441-w

**Published:** 2021-03-16

**Authors:** Gregory Simpson, Peter N. Thompson, Claude Saegerman, Tanguy Marcotty, Jean-Jacques Letesson, Xavier de Bolle, Jacques Godfroid

**Affiliations:** 1 Department of Production Animal Studies, Faculty of Veterinary Science, University of Pretoria, Onderstepoort, 0110 South Africa; 2 Centre for Veterinary Wildlife Studies, Faculty of Veterinary Science, University of Pretoria, Pretoria, South Africa; 3 Department of Infectious and Parasitic Diseases, Fundamental and Applied Research for Animal and Health (FARAH) Centre, Faculty of Veterinary Medicine, University of Liège, Liège, Belgium; 4 Faculty of Science, University of Namur, Namur, Belgium; 5 Department of Veterinary Tropical Diseases, Faculty of Veterinary Science, University of Pretoria, Pretoria, South Africa; 6 Department of Arctic and Marine Biology, Faculty of Biosciences, Fisheries and Economics, UiT, The Arctic University of Norway, Tromsø, Norway

**Keywords:** Infectious diseases, Bacteria, Conservation biology

## Abstract

This study aimed to consolidate current knowledge of wildlife brucellosis in Africa and to analyse available predictors of infection. The Preferred Reporting Items for Systematic Reviews and Meta-Analyses guidelines were followed. Information on species, test used, test results, area, rainfall, livestock and wildlife contact and year of study were extracted. This systematic review revealed 42 prevalence studies, nine disease control articles and six articles on epidemiology. *Brucella abortus, Brucella melitensis, Brucella inopinata* and *Brucella suis* were reported in wildlife. The prevalence studies revealed serological evidence of brucellosis in buffalo, antelope (positive in 14/28 species), carnivores (4/12) and other species (7/20) over the last five decades. Buffalo populations were more likely to be infected and had a higher seroprevalence than other species; the pooled seroprevalence was 13.7% (95% CI 10.3–17.3%) in buffalo, 7.1% (95% CI 1.1–15.5%) in carnivores and 2.1% (95% CI 0.1–4.9%) in antelope. Wildlife in high rainfall areas (≥ 800 mm) were more likely to be infected, and infected populations showed higher seroprevalence in high rainfall areas and in studies published after 2000. Domestic animal contact was associated with increased seroprevalence in antelope and carnivore species, but not in buffalo, supporting the hypothesis that buffalo may be a reservoir species.

## Introduction

Brucellosis caused by *Brucella* spp. is a disease of significant economic, public health and veterinary importance. Since its identification over 120 years ago in humans it has been isolated in wide variety of animals and found to have a global distribution. The main aetiological agents in humans are *Brucella abortus* and *Brucella melitensis*, which are predominantly carried by large and small ruminants respectively.

Brucellosis in wild animals in Africa has been documented in a variety of countries since the early 1960′s with serological studies and some *Brucella* isolations in many wildlife species^1^. Most studies have been serological surveys to try to better understand the epidemiological situation in wildlife, with an assumption that wildlife infected with *Brucella* spp. may have implications for domestic animals and humans. There has been no previous systematic review and meta-analysis of brucellosis in wildlife in Africa of this nature.

The objectives of this systematic review were to update answers to the following questions:

I. Which wildlife species have been exposed to brucellosis and where are they found?
II. Which *Brucella* species are known to infect wildlife species?
III. Which wildlife species are able to sustain *Brucella* infections?
IV. Are wildlife species a brucellosis risk to domestic animals and vice-versa?
V. What is known about effective control and prevention methods in wildlife?
VI. What factors are associated with brucellosis infection in wildlife?

## Results

### Inclusion criteria, quality control and data extraction

The titles and abstracts or the full articles were screened by the primary author to ensure that they met the following criteria:

I. The article appeared in a peer-reviewed journal and books. Conference proceedings and lay-media were excluded,
II. The article was for a study partially or fully conducted in Africa,
III. The article referred to brucellosis or *Brucella* spp.,
IV. The article involved wildlife (undomesticated animals living in the wild),
V. The article either provided information on prevalence, incidence or isolation of *Brucella* spp. or information on control, diagnosis or risk factors to brucellosis in wildlife in Africa.

**Figure 1:**
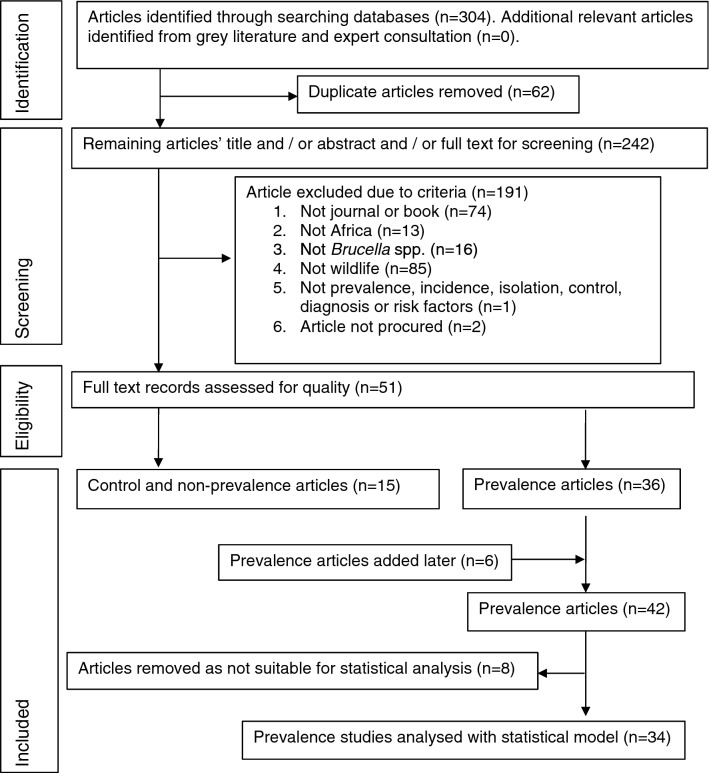
Flow diagram of study methodology.

62 duplicates were removed and screening with the above criteria removed 189 articles (74 did not meet criteria I, 13 did not meet criteria II, 16 did not meet criteria III, 85 did not meet criteria IV and one did not meet criteria V and two did not meet criteria VI) (Excluded articles in supplementary information). Two articles were not located. The remaining 51 articles, plus 6 additional articles^2–7^ located from references in the reviewed articles were separated into epidemiology (9), control (6), bacteriological (9) and prevalence (42) categories (Fig. 1). There was overlap with bacteriological and prevalence categories. The resulting articles were then reviewed and reported in the results section. Bacteriological results were reported separately.

The prevalence study articles were then assessed as to whether they had the following requirements for the meta-analysis:

I. The species, study sites, sample sizes and results were described.
II. The species were terrestrial mammals.
III. Serological testing was done and the test(s) described.
IV. Risk factors for brucellosis infection, such as livestock contact, was indicated or could be inferred.

Prevalence studies that did not meet the above requirements were not included in the meta-analysis. Eight of the above studies were removed before statistical analysis for the following reasons: the study was of fish^8^, dolphins^9^, rats^10^, no sample sizes were given^11–13^, no serological tests were done^14^ and one study used a card test not used by any other study^15^, leaving 34 studies for the meta-analysis. Rodents were excluded as there was only one study in this animal group, which tested wild rats and stray dogs that were co-habiting with humans and livestock^10^.

The remaining prevalence study articles had the following variables extracted for the statistical analysis: location, livestock contact (none, low degree, high degree, unknown), species, number of animals, year(s) of study, type of study, test(s) used and test results. The rainfall was calculated by using gridded mean annual precipitation data for the period ~ 1970–2000^16^ obtained at 10-min spatial resolution from www.worldclim.org (accessed 22 Sep 2017). Annual rainfall was recorded as the cell value for the location of the study if reported, or the mean of all the cell values contained within the geographic extent of the study location. For protected areas, shapefiles were obtained from www.protectedplanet.net (accessed 22 Sep 2017) and spatial overlays performed in ArcGIS 10.2 (Esri, Redlands, CA).

### Control and non-prevalence articles

The control and non-prevalence articles (9)^17–25^ and control articles (6)^26–31^ covered a variety of topics. Wildlife-related content of articles ranged from being limited, such as wildlife experts being involved in a qualitative general disease prioritization study with many non-wildlife experts^28^, to those only dealing with wildlife^17^. Only three of these 15 articles focused exclusively on brucellosis, of which one looked at management challenges across humans and domestic and wildlife species^26^ and two involved novel *Brucella* spp. in frogs^19,25^.

### Disease prioritization

A group of 36 veterinary, medical and wildlife health (n = 2) experts in Kenya ranked brucellosis the fourth most important zoonotic disease after anthrax, trypanosomiasis and rabies^28^. A semi-quantitative One Health Disease prioritization tool was used. Although the role of wildlife was not discussed in the article, endemic diseases in Africa, such as brucellosis, had high priorities due to their high prevalences. The wildlife-livestock interface is complex and challenging for human, animal and environmental health practitioners, with poorer communities carrying a higher risk of transmission to their animals from wildlife^31^. The need for veterinarians to monitor wildlife diseases, design control strategies and collaborate with other health care professionals was highlighted^31^.

### Host status

It is widely believed that *Brucella* spp. originated in livestock and spread to wildlife (spillover)^26^ and that *Brucella* spp. did not spill back to cattle in Africa^17^. Yet, wildlife in Africa is now seen as a source for re-emergence of the disease^30^. Studies performed in cattle in Uganda and Tanzania suggest that some *Brucella abortus* strains isolated from cattle have a genotype not found anywhere else^32,33^. Likewise, the reference *B. abortus* biovar 3 reference strain Tulya, isolated in Uganda from a human patient in 1959, is not representative of *B. abortus* biovar 3 strains isolated in other regions, particularly in Europe^34^. This suggests that a cluster of strains is circulating in cattle in some East African countries, whose origin remains elusive and for which an unknown wildlife reservoir may exist.

Certain wildlife species can also maintain the infection in the absence of contact with cattle. This maintenance in the absence of cattle has been suggested in bison (*Bison bison*) and elk (*Cervus canadensis*) in the Yellowstone Greater Conservation Area^35^. It has been suggested that brucellosis has established itself in Kafue lechwe antelope (*Kobus leche kafuensis*) in Zambia, which have a prevalence of 43% compared to 0% in black lechwe antelope (*Kobus leche smithemani*), where no contact with cattle has been noted^36^. Antelope are thought to play little role in dissemination of the disease to cattle compared to buffalo, but the disease does spread to some extent in antelope^24,36^. Yet, cattle interaction with Kafue lechwe was seen as a risk factor for cattle in Zambia^31^.

### Population impact

In wild African buffalo (*Syncerus caffer*) *Brucella abortus* biovar 1 causes abortions, but has limited impact^24,37^. A study found few in utero deaths in more than 3000 pregnant buffalo culled and the infected wild population showed a 12–15% annual increase in population size despite carnivore predation^17^. However, there is no knowledge of for how long evidence of foetal death would be detectable in buffalo, making it hard to interpret the in utero death findings. A more recent prevalence study found that buffalo serologically positive for brucellosis had lower body condition score, increased mortality and the population growth in infected herds was lower than uninfected herds^37^. The clinical severity of *Demodex* infection in the buffalo population in the Kruger National Park (KNP), South Africa, was also not associated with the presence of *Brucella* infection^23^. It is therefore premature to make a prediction on the long term impact of brucellosis in many wildlife populations^21^.

There has been a suggestion that gregarious wildlife species such as buffalo, eland (*Taurotragus oryx*), impala (*Aepyceros melampus*) and wildebeest (*Connochaetes taurinus*) had a higher seroprevalence than more solitary animals such as black (*Diceros bicornis*) and white (*Ceratotherium simum*) rhinoceros^26^. Although this is biologically plausible, no further support for this could be found.

### Novel species

A novel *Brucella* spp. was found in African bullfrogs (*Pyxicephalus edulis*) from Tanzania^19^. ‘Atypical’ *Brucella* spp. have been found in amphibians across the globe and the pathogenicity and significance is unknown^25^. They may only be commensals, but they do also cause severe pathology and could be a significant threat to amphibian populations^25^. Amphibians, even uncooked, are consumed by humans in Africa in large numbers^25^, and ‘atypical’ *Brucella* species with deviant lipopolysaccharide may impair serological diagnosis of these species in humans^25^.

### Vectors

The ticks *Hyalomma marginatum*, *Haemaphysalis punctata* and *Haemaphysalis sulcata*, which are known to harbour *Brucella melitensis*, were found on migrating birds in Egypt travelling from Africa^38^. However, the significance and role of ticks in disease transmission is unknown. This study however did not show evidence of the bacteria, but indicated its possible presence through the presence of the ticks.

### Control and testing

Attention should be given to livestock disease control amongst communities on the periphery of conservation areas due to the risk of spillover to wildlife^26^. More than 40% of farmers with a mixed farming system of wildlife and domestic animals surveyed in South Africa had no control measures to prevent interactions between the two groups of animals^30^. Specific gaps such as the establishment of health plans, routine health inspections, screening for zoonotic diseases, provision of quarantine camps and record keeping of health and withdrawal periods have been identified in game farming practices^30^.

Disease testing should be done in wildlife and livestock in close proximity to prevent transmission between the two groups and for wildlife when movement occurs and livestock when moving into an area with wildlife^21,27^. Ducrotoy et al.^26^ raised the need to validate diagnostic serological testing for wildlife. Serological testing for wildlife uses tests developed for domestic animals and are not validated for wildlife, which questions their validity in wildlife. However, there are currently no alternatives, which highlights the need to combine tests, the importance of strain isolation and the use of modern genetic tests for identification and classification, as well as the need to validate serological tests for various wildlife species, including camels^26^. Although there are registered vaccines for livestock, this is not the case in wildlife and their efficacy and safety is unknown^21^. For this reason, vaccination of wildlife is not undertaken in Africa.

The creation of large areas under transfrontier conservation initiatives have promoted sharing of ecological systems by humans, wildlife and domestic animals and may promote inter-species transmission of *Brucella* spp.^26^. The presence of *Brucella* spp. in domestic animals and wildlife compounds the public health risk, especially to resource poor communities living in this ecological setting^26^. However, the control in free-ranging wildlife is hardly practical, thus the control of brucellosis in domestic animals may be key to reducing the risk to humans. Community involvement and a “One Health” approach were also seen as necessary for disease control strategies^29^ including brucellosis control strategies. Surveillance of wildlife may need to be added to routine domestic animal surveillance as wildlife could be a potential direct source of infection for humans^39^, although this has not been demonstrated.

The transmission of *Brucella* spp. to humans via the consumption of bush meat and game meat is a potential public health risk^22^ and infected bush meat can be a risk to humans in shared ecosystems^39^. Yet, no human cases have been found in personnel, including veterinary, in the KNP where the disease is endemic in buffalo (*Syncerus caffer*)^18^ and staff consume buffalo meat. This study did not mention numbers of staff tested and if they had consumed meat or worked with buffalo so it is not possible to draw a correlation here. Yet, in Tanzania in 1969 impala handling was implicated in infecting three personnel^3^. The risk through consumption of infected meat is unknown and often speculated, but it would be valuable to quantify.

### Bacteriological studies

We found nine studies detailing the identification of *Brucella* spp. from across the continent (Table 1). They were from a variety of wildlife including buffalo, antelope, wild rats (and dogs in this study), fish and frog. This diversity of animals harbouring the bacteria indicates infection is widespread. Four different species *B. suis*, *B. abortus, B. melitensis* and *B. inopinata* and five different biovars *B. suis* (biovar 3), *B. abortus* (biovars 1 and 3) and *B. melitensis* (biovars 1 and 3) were identified. All the studies except for two were done before 1978, indicating that very few isolation studies have been published and even fewer in the last decade.

**Table 1:** *Brucella* spp. isolation studies in Africa with biovar, year of study, country, method of identification and references.

Species	Country	Year	Brucella	Biovar	Identification	References
Rodents	Kenya	1963	*B. suis*	3 *	Culture	^72^
Buffalo (*Syncerus caffer*)	Tanzania	1969	*B. abortus*	3	Agglutination, complement fixation and culture (guinea pig and CO_2_)	^2^
Impala (*Aepyceros melampus*)	Tanzania	1971	*B. melitensis*	1	Agglutination (1:40 cutoff) and culture (guinea pig)	^3^
Wild rats	Egypt	1974	*B. abortus*	3	Agglutination (1:40 cutoff) and culture (CO_2_)	^10^
Buffalo (*Syncerus caffer*)	South Africa	1977	*B. abortus*	1	Impression smear and culture (CO_2_)	^14^
Waterbuck (*Kobus ellipsiprymnus*)	Zimbabwe	1969	*B. abortus*	1	Impression smear and culture (CO_2_)	^12^
Eland (*Tragelaphus ory*x)	Zimbabwe	1972	*B. abortus*	1	Agglutination and culture (guinea pig)	^47^
Nile catfish (*Clarias gariepinus*)	Egypt	2008	*B. melitensis*	3	Agglutination, Rivanol, culture (CO_2_) and PCR	^8^
African bullfrog (*Pyxicephalus edulis*)	Tanzania	2012	*B. inopinata*		Agglutination, culture (CO_2_), CFT, PCR, MLSA	^19^

*CFT* complement fixation test, *PCR* polymerase chain reaction, *MLSA* multilocus sequence analysis.

*Possible misclassification because the species and the biovar were ascribed by phenotypic characterization only.

### Spatial distribution of prevalence study results

The studies were predominantly conducted in southern and eastern Africa, with the most being in Zimbabwe (10), South Africa (9) and Tanzania (7) (Table 2). Notably, no studies were found addressing brucellosis in wildlife in North and West Africa.

**Table 2:** Prevalence studies on brucellosis in African wildlife by country in decreasing order with associated region (according to the African Union).

Country	No of studies	Region	References
Zimbabwe	10	Southern	^4,11,12,41,47,73–77^
South Africa	9	Southern	^6,9,14,37,78–82^
Tanzania	7	Eastern	^2,3,7,83–86^
Zambia	4	Southern	^13,59,87,88^
Kenya	3	Eastern	^79,89,90^
Namibia	3	Southern	^79,91,92^
Botswana	2	Southern	^39,93^
Egypt	2	North	^8,10^
Uganda	2	Eastern	^5,94^
Democratic Republic of Congo	1	Central	^15^
Mozambique	1	Southern	^95^

### Quality analysis of prevalence studies

Three questions of the appraisal tool^40^ were deemed not applicable, namely (2) Were study participants recruited in an appropriate way? (5) Is the data analysis conducted with sufficient coverage of the identified sample? (9) Are all important confounding factors/ subgroups/differences identified and accounted for?

The results for the remaining questions (Table 3) showed that in general the published studies used small sample sizes and it was unclear whether the samples were representative of the target population.

**Table 3:** Results of appraisal tool questions for prevalence studies.

	Was the sample representative of the target population?	Was the sample size adequate?	Were the study subjects and setting described in detail?	Were objective, standard criteria used for measurement of the condition?	Was the condition measured reliably?	Was there appropriate statistical analysis?	Were subpopulations identified using objective criteria?
Yes	38%	47%	47%	100%	79%	35%	44%
No	9%	41%	53%	0%	0%	65%	50%
Unclear	53%	12%	0%	0%	21%	0%	6%

### Statistical analysis of prevalence studies

We found buffalo, 28 antelope, 12 carnivore and 20 other species tested for brucellosis in the selected prevalence studies (Table 4). The prevalence of positive results varied from zero to 100%; however, all of the latter only had one animal in the study. Buffalo had the greatest number of studies per species at 24, followed by 12 for impala, 10 for blue wildebeest (*Connochaetes taurinus)* and 8 for giraffe (*Giraffa camelopardalis*). The studies used the Rose Bengal test (RBT), serum agglutination test (SAT), complement fixation test (CFT), indirect and competitive enzyme linked immunosorbent assays (ELISA) and fluorescence polarization assay (FPA). For the purpose of statistical analysis, the confirmatory test was used as the test type. There were no studies that used RBT as the only test.

**Table 4:** Range of results for serological prevalence of *Brucella* spp. in African wildlife by species arranged in groups: buffalo, antelope, carnivores and all other species.

Buffalo	No studies	Seroprevalence range (%)	References
Buffalo (*Syncerus caffer*)	24	0–53	^2,4–7,37,39,41,73–76,78,80,83–86,89,90,93,95–97^
**Antelope**			
Black lechwe (*Kobus leche smithemani*)	1	0	^36^
Black-faced impala (*Aepyceros melampus petersi*)	1	0	^92^
Blesbok (*Damaliscus dorcas phillipsi)*	1	0	^41^
Blue wildebeest (*Connochaetes taurinus*)	10	0–27.3	^6,7,39,41,47,74,85,86,90,93^
Bushbuck (*Tragelaphus scriptus*)	5	0–10	^39,41,47,74,93^
Dik dik (*Rhynchotragus kirkii*)	1	0	^85^
Duiker (*Silvicapra grimmia*)	2	0–2.7	^47,74^
Eland (*Taurotragus oryx*)	6	0–27.3	^5,7,39,47,74,85^
Gemsbok (*Oryx gazella)*	1	0	^39^
Grant's gazelle (*Nanger granti*)	2	0	^7,85^
Grysbok (*Raphicerus sharpei*)	1	4	^74^
Hartebeest (*Alcelaphus buselaphus*)	3	0	^39,85,93^
Impala (*Aepyceros melampus*)	12	0–11.5	^3,4,6,7,39,41,47,73,74,85,93,96^
Kafue lechwe (*Kobus leche kafuensis*)	3	10–42.3	^36,59,88^
Klipspringer (*Oreotragus oreotragus*)	1	0	^47^
Kudu (*Tragelaphus strepsiceros*)	8	0–3	^4,6,39,41,47,74,93,96^
Lechwe (*Kobus leche*)	2	0	^39,93^
Nyala (*Tragelaphus angasi*)	2	0	^41,47^
Reedbuck (*Redunca arundinum*)	5	0	^39,41,47,74,85^
Roan (*Hippotragus equinus*)	3	0	^6,74,85^
Sable (*Hippotragus niger*)	4	0–5.2	^6,41,47,74^
Springbok (*Antidorcas marsupialis)*	3	0	^39,91,93^
Steenbok (*Raphicerus campestris*)	3	0	^39,47,74^
Suni (*Nesotragus moschatus*)	1	0	^47^
Thomson's gazelle (*Eudorcas thomsonii*)	2	0–2.4	^7,85^
Topi (*Damaliscus korrigum*)	2	2.3	^7,85^
Tsessebe (*Damaliscus lunatus*)	5	0–11	^6,41,47,74,93^
Waterbuck (*Kobus ellipsiprymnus*)	7	0–100	^6,7,39,41,47,74,85^
**Carnivores**			
Bat-eared fox (*Otocyon megalotis)*	1	0	^85^
Banded mongoose (*Mungos mungo)*	1	0	^85^
Black-backed jackal (*Canis mesomelas*)	1	42.9	^85^
Civets and genets (Viverridae)	1	0	^74^
Genet cat (*Genetta genetta*)	2	0	^47,85^
Honey badger (*Mellivora capensis)*	1	0	^39^
Leopard (*Panthera pardus*)	1	0	^85^
Lion (*Panthera leo*)	5	0–50	^6,39,41,84,85^
Serval (*Felis serval)*	1	0	^41^
Spotted hyaena (*Crocuta crocuta*)	3	0–50	^6,7,85^
White-tailed mongoose (*Ichneumia albicauda*)	1	0	^85^
Wild dog (*Lycaon pictus)*	1	33.3	^85^
**Others**			
Antbear (*Orycteropus afer*)	2	0	^47,74^
Baboon (*Papio ursinus*)	5	0–27	^6,47,82,85,89^
Black rhinoceros (*Diceros bicornis)*	3	0–6.3	^73,74,79^
Bottlenose & Indo-pacific humpback dolphin (*Tursiops aduncus* & *Sousa plumbea*)	1	0	^9^
Bushpig (*Potamachoerus porcus*)	1	0	^47^
East African hare (*Lepus capensis*)	1	0	^85^
Elephant (*Loxodonta africana*)	6	0	^5,6,39,41,47,74^
Giraffe (*Giraffa camelopardalis*)	8	0–100	^5,6,39,41,47,74,77,85^
Hippopotamus (*Hippopotamus amphibius*)	4	11.1–25.5	^5,6,47,94^
Jumping hare (*Pedetes surdaster*)	1	0	^85^
Nile catfish (*Clarias gariepinus*)	1	9.2	^8^
Porcupine (*Hystrix Africae-Australis*)	1	0	^47^
Primates (*Papio* spp.*, Cercopithecus* spp.)	1	0	^74^
Rockrabbit (*Procavia capensis*)	1	0	^74^
Rodents (*Pedetes, Lepus, Hystrix* spp.)	1	0	^74^
Spring hare (*Pedetes capensis*)	1	0	^47^
Vervet monkey (*Cercopithecus aethiops*)	2	0	^6,41^
Warthog (*Phacochoerus aethiopicus*)	6	0–1.5	^39,41,47,74,85,93^
White rhinoceros (*Ceratotherium simum*)	5	0	^6,39,47,73,79^
Zebra (*Equus quagga burchelli*)	8	0–100	^5,6,39,41,47,74,84,85^

### Univariate meta-analysis of prevalence

Not all of the prevalence studies were included in the statistical analysis (34/42). The overall pooled estimate of seroprevalence in all wildlife species combined was 4.6% (95% CI 2.2–7.4%), with high heterogeneity (*I*^2^ = 87%; *P* < 0.001). Pooled estimates of seroprevalence were highest in buffalo and lowest in antelope, and heterogeneity was high in all species categories except in carnivores (Table 5). Overall, the study that had the greatest weight and influence in the analysis was the report by Madsen^41^ of 1920 impala in Zimbabwe that all tested negative; omission of this study from the analysis resulted in a pooled prevalence estimate of 6.0% overall and 3.4% in antelope.

**Table 5:** Pooled prevalence and heterogeneity estimates in a meta-analysis of prevalence of brucellosis in African wildlife species.

Species	No. of prevalence reports	Pooled prevalence (%)	95% CI	Heterogeneity	
				Higgins’ *I*^2^	*P*-value
Buffalo	65	13.7	10.3–17.3	82%	< 0.001
Antelope	115	2.1	0.1–4.9	85%	< 0.001
Carnivores	19	7.1	1.1–15.5	28%	0.130
Other	72	2.8	0.6–5.6	74%	< 0.001
Total	271	4.6	2.2–7.4	87%	< 0.001

**Figure 2:**
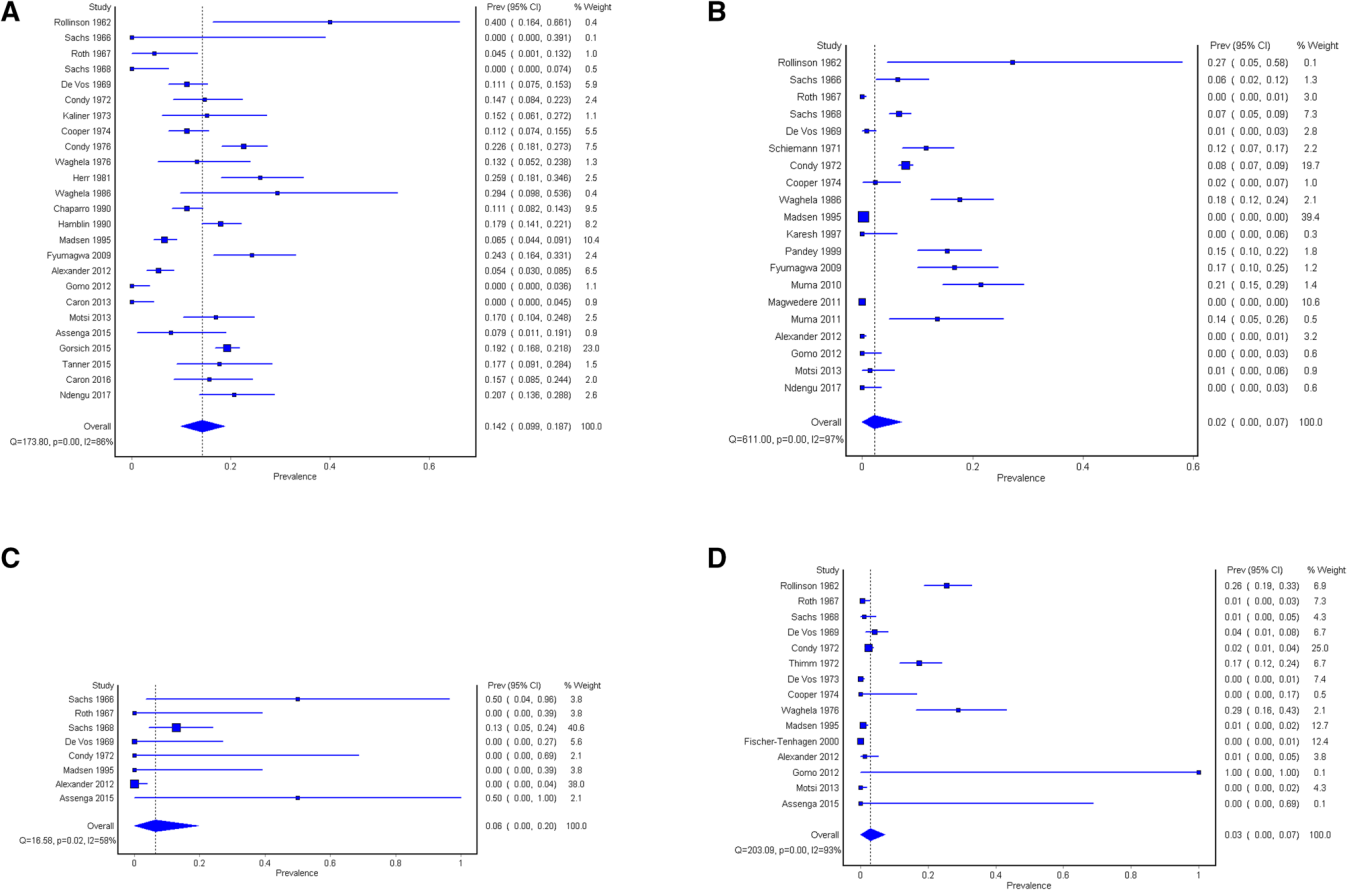
(**a**) African buffalo. (**b**) African wild antelope species. (**c**) African wild carnivore species. (**d**) African wildlife species excluding buffalo, antelope and carnivores. Forest plots of *Brucella* seroprevalence in: (**a**) African buffalo (n = 25 studies), (**b**) African wild antelope species (n = 20 studies), (**c**) African wild carnivore species (n = 8 studies) and (**d**) African wildlife excluding buffalo, antelope and carnivores (n = 15 studies), aggregated by publication, with weight contribution to pooled prevalence estimate (proportional to the inverse variance of each study’s prevalence estimate). Studies are shown chronologically from top to bottom. Blue squares show point estimates, with size of square proportional to sample size; horizontal lines indicate 95% confidence intervals; diamond shows point estimate and 95% confidence interval for pooled estimate. I2 is the heterogeneity (Higgins’ *I*^2^), indicating the proportion of variability between study results attributed to heterogeneity. Q is the Cochran’s statistic used to test the null hypothesis that *I*^2^ = 0.

Seroprevalences aggregated by publication and in chronological order, are shown for African buffalo in Fig. 2a, for antelope species in Fig. 2b, for carnivore species in Fig. 2c and for other species in Fig. 2d. The pooled prevalence and heterogeneity estimates shown in the forest plots differ slightly from those in Table 6 as sub-studies within a publication were combined for brevity.

**Table 6:** Meta-regression model of factors associated with seropositivity to *Brucella* in published studies of African wildlife, using a zero-inflated negative binomial model with robust variance estimates.

Variable and category	Parameter estimate	95% confidence interval	*P*-value
Count model (seroprevalence within infected population)	Count ratio*		
**Species**			
Buffalo	1†	–	–
Antelope	0.42	0.25–0.68	0.001
Carnivore	1.19	0.53–2.71	0.673
Other	0.92	0.47–1.78	0.796
**Year**			
< 1980	1†	–	–
1980–2000	1.38	0.84–2.28	0.208
> 2000	1.73	1.17–2.56	0.006
**Annual rainfall (mm)**			
< 500	1†	–	–
500–599	1.25	0.84–1.85	0.271
600–799	1.31	0.92–1.89	0.138
≥ 800	1.82	1.10–3.02	0.019
**Serological test**			
SAT	1†	–	–
CFT	0.79	0.51–1.24	0.306
ELISA	1.06	0.64–1.75	0.815
FPA	0.36	0.19–0.68	0.001
Inflation model (Odds of population being non-infected)	Odds ratio‡		
**Species**			
Buffalo	1†	–	–
Antelope	14.8	3.06–71.3	0.001
Carnivore	62.7	3.23–> 10^3^	0.006
Other	43.8	6.18–310	< 0.001
**Year**			
< 1980	1†	–	–
1980–2000	2.26	0.43–11.8	0.334
> 2000	0.71	0.09–5.30	0.736
**Annual rainfall (mm)**			
< 500	1†	–	–
500–599	0.25	0.03–1.82	0.171
600–799	0.51	0.11–2.48	0.405
≥ 800	0.07	0.00–0.91	0.043
**Serological test**			
AT	1†	–	–
CFT	3.45	0.77–15.4	0.104
ELISA	6.03	1.21–29.9	0.028
FPA	0.73	0.06–8.47	0.802

*α* (overdispersion parameter) = 0.37 (95% Confidence interval: 0.21–0.66; *P* = 0.001); Akaike’s information criterion = 804.

*Ratio of seroprevalence vs. reference category, within infected population. † Reference category.

^‡^Ratio of odds of population being non-infected vs. reference category.

**Figure 3:**
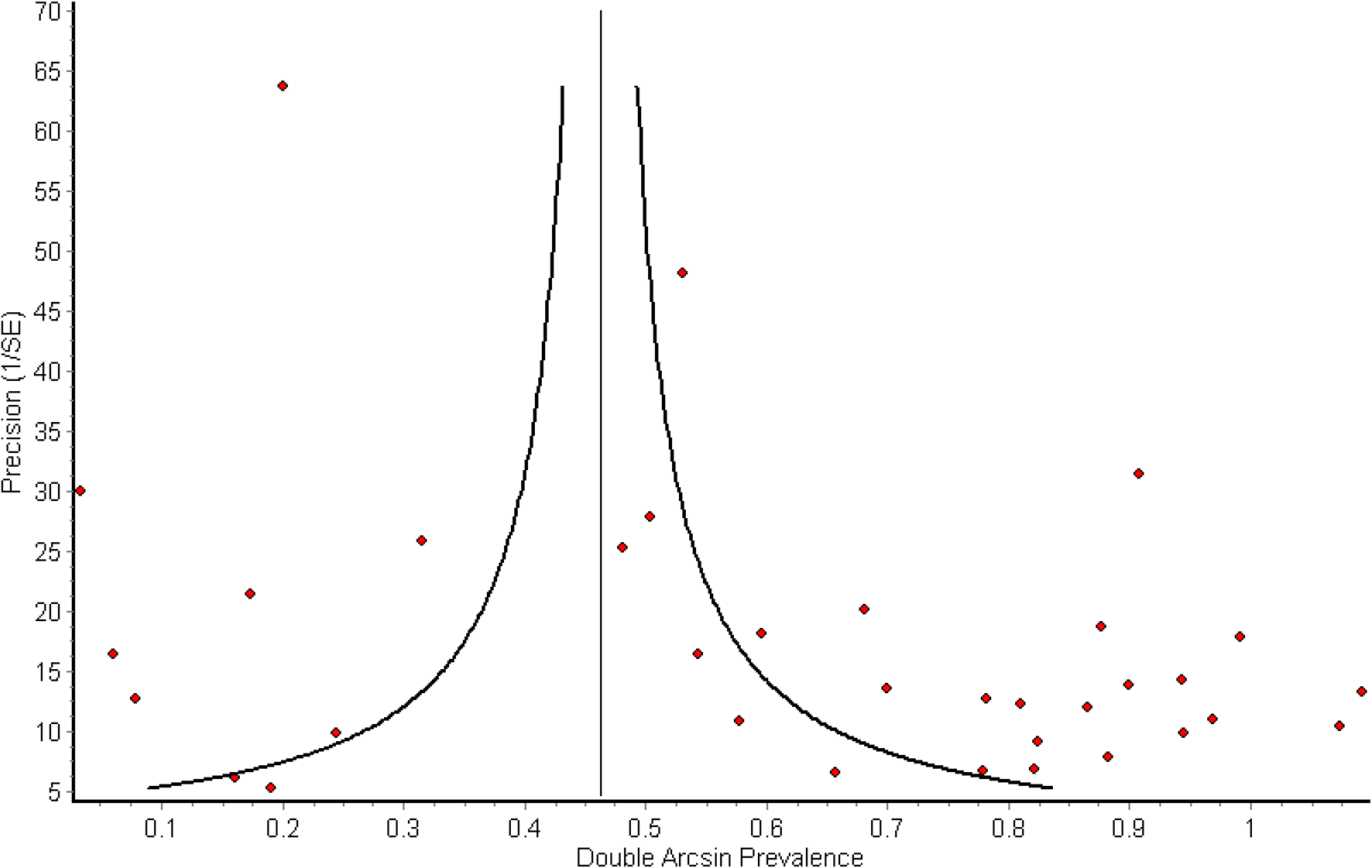
Funnel plot of published studies (n = 34) on *Brucella* seroprevalence in African wildlife showing study precision vs. transformed prevalence estimate. Curved lines indicate cut-off for statistically significant difference (*P* < 0.05) vs. pooled estimate (vertical line).

The funnel plot for all publications combined (Fig. 3) shows marked asymmetry, with lower precision, i.e. smaller studies tending to show higher prevalence estimates. This is most likely due to publication bias, with smaller studies showing “negative” results less likely to have been published. This was seen in all species categories except buffalo, where only very minor asymmetry was observed, suggesting that studies performed on buffalo were likely to be published irrespective of outcome. The plot also shows a large horizontal spread of points due to high heterogeneity.

### Multivariable meta-regression of selected prevalence studies

In the zero-inflated negative binomial meta-regression model (Table 6), the count model assessed factors associated with seroprevalence within infected populations, while the inflation model assessed factors associated with the population being non-infected. Several factors were associated both with the likelihood of a population being infected and with the seroprevalence within infected populations. The significance of the overdispersion parameter confirmed the suitability of the negative binomial vs. Poisson model and the AIC of 804 vs. 841 for the negative binomial model confirmed the suitability of the zero-inflated model. Note that the odds ratios (OR) in the inflation model in Table 5 refer to the odds of being negative, therefore their reciprocal, the odds ratios for being infected, are quoted in the summary below. In addition, the interaction between species and degree of livestock contact was significant in the count model (*P* = 0.041), therefore the association of livestock contact with seroprevalence is shown separately for each species in Table 6.

Buffalo populations were the most likely to be infected, with antelope (OR = 0.07; *P* = 0.001), carnivores (OR = 0.01; *P* = 0.006) and other species (OR = 0.02; *P* < 0.001) less likely to be infected. Within infected populations, antelope showed a lower seroprevalence than buffalo (OR = 0.42; *P* = 0.001).

Seroprevalence appeared to increase over time, being significantly higher after 2000 than pre-1980 (OR = 1.73; *P* = 0.006). Although year was not significant in the inflation model (*P* = 0.137), it was retained as a confounder, and populations did tend to be more likely to test positive post-2000 than during 1980–2000 (OR = 3.19; 95% CI 0.90–11.3; *P* = 0.071). Seroprevalence also increased with rainfall; in areas with annual rainfall > 800 mm populations were more likely to be infected (OR = 14.3; *P* = 0.043) and the seroprevalence was higher (OR = 1.82; *P* = 0.019). The type of diagnostic test used was a significant source of variation in both components of the model (*P* = 0.022); however, its inclusion was primarily in order to control for confounding.

The degree of livestock contact was associated with seroprevalence in antelope and carnivore species (Table 7), with a high degree of contact associated with the highest seroprevalence in antelope (OR = 10.9; *P* = 0.001). However, this was not seen in buffalo or in other species.

**Table 7:** Association between degree of livestock contact and seroprevalence to *Brucella* in different wildlife species categories, obtained from the count model presented in Table 6.

Level of livestock contact	Count ratio*	95% confidence interval	*P*-value
**Buffalo**			
None	1†	–	–
Low	0.92	0.49–1.71	0.781
High	1.05	0.63–1.75	0.863
Unknown	1.09	0.61–1.96	0.763
**Antelope**			
None	1†	–	–
Low	6.53	1.47–29.1	0.014
High	10.9	2.37–50.4	0.002
Unknown	8.25	1.86–36.6	0.006
**Carnivore**			
None	1†	–	–
Low	> 10^3^	> 10^3^–> 10^3^	< 0.001
High	> 10^3^	> 10^3^–> 10^3^	< 0.001
Unknown	0.25	0.02–3.04	0.278
**Other**			
None	1†	–	–
Low	1.24	0.36–4.34	0.733
High	0.17	0.03–0.96	0.045
Unknown	2.68	1.16–6.20	0.021

*Ratio of seroprevalence vs. reference category, within infected population.

^†^Reference category.

## Discussion

This systematic review and meta-analysis aimed to collate the knowledge on which African wildlife species have been infected with brucellosis, by which *Brucella* species, which wildlife species are able to sustain *Brucella* infections, which wildlife species can be reservoirs and what factors are associated with *Brucella* infection and seroprevalence in wildlife. A large number of wildlife species have been found to be infected or to show serological evidence of exposure to *Brucella* spp. Multiple species can show evidence of infection, which could result in dilution or amplification of prevalence as suggested with increased biodiversity^42^. This has not been investigated in wildlife in Africa, nor have the composition and dynamics of reservoir systems, which can consist of a species, population or maintenance community^43^.The ability to maintain a sustainable infection within a given wildlife species or population without exposure from other sources has often not been addressed. In addition, if a wildlife population sustains a *Brucella* infection it does not mean it will transmit it to other species as that depends on many factors such as whether abortions occur, behaviour during parturition and management practices. Finally, the ability of gaining or losing maintenance capacity has been documented to be driven by changing ecological systems^42^.

The potential impact of infected wildlife on public health depends thus primarily on *Brucella* sustainability (spillover versus reservoir host) and prevalence in a given wildlife species. Human activities related to infected wildlife species such as hunting, dressing of carcasses, meat handling, consumption, wildlife sampling and management in more intensive settings contribute to transmission of the infection. Disease control must be focused where the greatest health benefit will be attained such as has been suggested with focused spatio-temporal vaccination for foot and mouth disease in Niger^44^. Vaccination can only be recommended for reservoir species, not for spillover species. In the African context, only vaccination in buffalo should be considered. However, this is not an option currently as there is no vaccine available for brucellosis in wildlife^45^. Therefore the focus should be on vaccination in domestic animals, testing and slaughter of infected domestic animals. The spatial and temporal separation management between wildlife and livestock is a sound management practice as highlighted in the Greater Yellowstone Area, USA^35^.

The non-English and grey literature searches and expert consultations did not add relevant information to the initial database search. Such findings have also been reported for the use of grey literature (non-English reports, unpublished studies and dissertations) in the analysis of child-relevant systematic reviews^46^. There was large variation in the quality of the prevalence studies with respect to the reporting of precise and reliable estimates of seroprevalence in their respective target populations and it is possible that the risk of bias in many of the studies was high. There were a few studies where representative sampling was done on populations, but in general it is very difficult to ensure representation in sampling from wildlife populations.

Investigation of exposure to *Brucella* spp. in wildlife species has been first done by serology. We must bear in mind that the antibody response to infection in certain wildlife species in this review was shorter and less significant in comparison to cattle^47^. This could mean an underestimation in wildlife in comparison to cattle. The RBT is a simple and reliable serological test recommended by the World Organisation for Animal Health (OIE). However, when using it in wildlife, one is confronted with two problems interfering with the partly subjective reading of the agglutination, which may lead to a biased result: (1) the presence of haemolysis (difficult for reading) and (2) fat globules wrongly identified as agglutinates in the sera. Moreover, some false positive serological reactions can also be observed in agglutination tests^48^. A recommended testing strategy for brucellosis in livestock is using a screening test (such as the RBT), followed by a confirmatory test (such as CFT or ELISA) performed on sera classified positive by the screening test (series testing), which was done in most of the prevalence studies. Nevertheless, the lack of validated diagnostic tests for *Brucella* spp. exposure in wildlife species remains a major limitation for this and any other study on the subject. The validation of tests for wildlife species is likely not feasible because of costs and logistics; therefore, alternative methods should be found. For example, serological studies for which only RBT has been used should always be analysed with caution and RBT should rather be compared to ELISA results and if there are large discrepancies between results in both tests, a chloroform/centrifugation cleaning up of sera should be performed prior to RBT testing^49^. Serological studies only inform on possible exposure to *Brucella* spp. In order to identify which *Brucella* species is responsible for seroconversion, isolation of *Brucella* spp. or its DNA identification is necessary, which was only performed in one study^19^. Molecular analyses should be performed for strains isolated from African wildlife as they will inform on the origin, the epidemiology and the transmission of *Brucella* spp. within and between different species. However, the veracity of this information is dependent on regular molecular typing of the circulating strains of *Brucella* spp., as demonstrated in the USA^35^.

The large variation in reported seroprevalence between studies was reflected in the high heterogeneity estimates. Although the grouping of multiple species into each species category may have contributed to heterogeneity, particularly in the Antelope and Other categories, heterogeneity was also high in buffalo, suggesting that factors other than species were largely responsible for this variation. Although our meta-analysis allowed us to estimate a pooled prevalence for each species group, its primary aim was rather to assess the factors responsible for heterogeneity, i.e. the varying determinants of seropositivity among individuals as well as populations. The zero-inflated negative binomial model allowed us to do this while correctly weighting the contribution of each study, while the robust variance estimates accounted for the clustering of multiple studies within publications. The seroprevalence within infected populations was significantly higher in studies after the year 2000 as compared to before 1980. This could be due to recent spread of the disease in wildlife populations or recent research focusing more on infected populations; however, a possible increase in publication bias over time, with positive studies more likely to be published, could also partly account for this. The areas of higher rainfall, over 800 mm, showed a greater likelihood of populations being infected with brucellosis and having a higher prevalence, which could be due to higher rainfall leading to more available grazing and increased population densities with a resultant increased pathogen transmission. *Brucella* seropositivity in cattle in Zimbabwe was found to be progressively higher with increasing stocking density and herd size^50^. In Uganda, a bimodal increase in prevalence was found in livestock associated with the rainfall seasons^51^. This is thought to be due to the calving periods that occur during this time and hence the increased presence of the bacteria due to parturition material and milk. The association with rainfall could also possibly be due to the fact that the bacteria survive longer in the environment in colder, wetter conditions with less sunlight, as found in bison (*Bison bison*) in the USA^52^. This factor should be considered when designing and implementing disease control programs.

Dry seasons resulting in increased densities or animals, both wild and domestic, around water sources may have a positive influence in transmission within and between species^39^. We found individual studies showing that proximity to wildlife reserves and porous nature of fences of wildlife reserves to be significant risk factors for brucellosis in cattle^4,53^. In our meta-analysis livestock contact was found to be a significant risk factor for brucellosis infection in antelope and carnivore species, but not buffalo. This suggests that buffalo may sustain *B. abortus* infections within infected herds without transmission from other sources. We found less evidence of publication bias for buffalo than in other species, i.e. in buffalo serological studies for *Brucella* spp. were likely to be published irrespective of outcome, whereas in other species there was evidence that positive serological findings were more likely to be published than negative ones. This could be due to the particular research interest in buffalo due to controlled diseases such as foot and mouth disease and tuberculosis for which they are reservoir hosts. Four of the seven studies of only buffalo were on multiple diseases, whereas all of the other 17 studies that included buffalo were only on brucellosis.

This review highlights that exposure to *Brucella* spp. has occurred in several species. However, only a handful of studies addressed the question of sustainability of *Brucella* infection in a given host species. Species that cannot sustain the infection without recurrent contact with an external source are called spillover species^54^. Sustainable infections have been described in buffalo and bison (*B. abortus*), Alpine ibex (*B. melitensis*)*,* wild boar and European hare (*Lepus europeaus*) (*B. suis* biovar 2), reindeer (*Rangifer tarandus*) (*B. suis* biovar 4), and cetaceans (*B. ceti*). This question remains to be addressed for the newly recognized *Brucella* species, i.e., *Brucella inopinata* in frogs and humans, *Brucella microti* in voles and red fox (*Vulpes vulpes*), *Brucella vulpis* in red fox and *Brucella papionis* in baboons (*Papio* spp.). It is of the utmost importance to be able to assess whether a wildlife species is a reservoir or a spillover host, as this has important implications for the control of the disease^55^. Indeed, control measures should always be first implemented, if possible, in reservoir species, not in spillover species^56^. Red deer (*Cervus elaphus*) is only a spillover host, rarely exposed to *B. abortus* infection in Europe, whereas the elk (*Cervus canadensis*) is a maintenance host in the Yellowstone Greater Conservation Area and has now replaced bison as a source of infection for cattle^57^. Spillback infection from elk to cattle is now of great concern and a cause of controversy between wildlife managers, hunters and livestock owners. As there is currently no vaccine registered for wildlife, management is currently based on spatio-temporal segregation between bison, elk and cattle^57^.

There is strong evidence that buffalo is a reservoir host for *B. abortus*^37^. Although *B. abortus* infection has a significant impact on individual animals, it is not considered a direct threat to the sustainability of buffalo herds in Kruger National Park, South Africa^17,37^. In a maintenance host like buffalo, higher within-herd prevalence could be due to their herd dynamics of having large herds that maintain close contact within the group in comparison to other species. Bovine tuberculosis is known to spread well between buffalo due to their social nature and large herd sizes, an average of 250 per herd in the Kruger National Park^58^. This is likely also to be the case for *B. abortus* infection. It remains to be determined whether other wildlife species besides buffalo are maintenance hosts, as suggested for Kafue lechwe^59^, and if so whether they can be potential sources of infection for other wildlife and livestock^54^.

A limitation of the study was the lack of uniformity in assessing the degree of contact with livestock between the different studies, and in many cases this could not be determined with any degree of certainty. Ideally, in the future studies should be conducted with variables that could be compared easily, e.g. distance between domestic and wildlife species and settings well explained, the presence of functional fences between wildlife and livestock, and vaccination practices in livestock. Another limitation is that the results compared were published over a 50 year period, which could have seen changes in laboratory practices. A further limitation was that most of the publications were from southern and eastern Africa, which leaves gaps in the knowledge for the whole continent. Also, smaller studies showing negative serological results are less likely to be published, except in the case of buffalo. It would be of benefit for more negative studies and studies from West and North Africa to be published to give a more complete understanding of the epidemiological situation on the African continent.

There is a dearth of knowledge of brucellosis in domestic and wild suids in Africa, with none of the latter having been found. Although it is thought that *Brucella suis* is prevalent in suids in Africa, its isolation from suids in sub-Saharan Africa has not been reported in the scientific literature^60^. Interestingly, it has been reported in cattle^61^ and swine^62^ in Egypt and in cattle in Zimbabwe^63^. *Brucella suis* is not a sustainable infection in cattle and the source of the infection has still to be identified in either domestic, wild or feral suids^33,64^. Lastly, new *Brucella* species have been described and the importance of *Brucella* exposure in marine mammals^65^, ectotherms such as frogs and fish^8,19^ and primates such as baboons^66^ needs to be assessed in the African context.

## Conclusion

Exposure to *Brucella* spp. has been detected by serological studies in a variety of wildlife species and brucellosis has been identified through culture in only a few wildlife species in Africa over the last five decades. Other continents have isolated *Brucella* spp. in a great variety of wildlife species including marine animals^67^. Shortcomings and lack of validation of brucellosis serological tests are important limitations in assessing the studies of this review, particularly if numbers of animals tested are limited. The number of published studies on brucellosis in Africa has increased markedly since 2010, indicating a growing interest on brucellosis in wildlife. Buffalo populations were more likely to be infected than any other species and showed higher prevalences. Epidemiological, serological and bacteriological evidences indicate that buffalo are a reservoir species and are able to sustain a *B. abortus* infection. Consequently, livestock contact was a predictor for brucellosis exposure in antelope and carnivores (spillover species), but not in buffalo (reservoir species). As human population growth drives wildlife habitat loss and increased overlap between domestic animals and wildlife, understanding brucellosis in wildlife and its potentially changing epidemiology is of increasing importance. Future research on brucellosis in Africa, especially in West and North Africa, should include the isolation, identification and molecular characterization of *Brucella* spp., as well as the changes in ecological ecosystems, to understand the origin, transmission patterns and drivers of infection in wildlife.

## Methods

### Systematic review protocol

The guidelines made by the Preferred Reporting Items for Systematic Reviews and Meta-Analyses (PRISMA) were followed. See supplementary material.

### Literature search and data collection

We searched the Web of Science (1910–June 2017), Scopus (1823–June 2017), Cochrane library (June 2017), Google Scholar (June 2017), Africa-wide Info (June 2017) and Wildlife and Ecology Studies Worldwide (June 2017). We used the Boolean Operators “or” and “and” for our search and used the following terms, in free word text and topic or subject heading:

I. “(Brucella OR brucellosis) AND
II. (wildlife OR wild) AND
III. Africa”.

No time limits were set. The databases search revealed 304 articles. The Web of Science (237), Scopus (20), Cochrane library (0), Africa-wide info (42) and Wildlife and ecology studies worldwide (5) (Fig. 3). There were no language restrictions and the search was also conducted in French, German and Portuguese.

Grey literature searches were done on OpenGrey: System for Information on Grey Literature in Europe (http://www.opengrey.eu/), OpenDOAR: The Directory of Open Access Repositories (https://v2.sherpa.ac.uk/opendoar/) and the OAIster database (https://www.oclc.org/en/oaister.html). Experts in Germany and Brazil were consulted for any additional articles of relevance published in German or Portuguese. Search results were managed with Excel and Mendeley.

### Prevalence study quality appraisal

The studies were evaluated using an appraisal tool^40^ designed for systematic reviews addressing questions of prevalence. The tool was made up of ten questions asked of each study: (1) Was the sample representative of the target population? (2) Were study participants recruited in an appropriate way? (3) Was the sample size adequate? (4) Were the study subjects and setting described in detail? (5) Is the data analysis conducted with sufficient coverage of the identified sample? (6) Were objective, standard criteria used for measurement of the condition? (7) Was the condition measured reliably? (8) Was there appropriate statistical analysis? (9) Are all important confounding factors/subgroups/differences identified and accounted for? (10) Were subpopulations identified using objective criteria?

### Statistical analysis

Grouped data were used for analysis, where a group represented data for a particular species and location that could be identified from a publication. Some publications therefore yielded several groups, each of which was therefore regarded as a separate study to be included in the meta-analysis. Meta-analysis of prevalence was done for all groups combined, as well as separately for each species category, using the double arcsine transformation^68^ in an inverse variance heterogeneity model, implemented in the MetaXL 5.3 add-in for Excel^69^. In this model, each study is weighted by the inverse variance of its prevalence estimate, but the variance of the pooled estimate is inflated to account for the heterogeneity^70^. Heterogeneity of estimates was assessed using the Higgins *I*^2^ statistic^71^, with *I*^2^ > 75% indicating high heterogeneity, and statistical significance of heterogeneity was assessed using the Cochrane’s Q statistic. In order to visually represent the data using forest plots, multiple groups of the same species category within a publication were aggregated and separate meta-analyses of prevalence were done for each species category. Assessment of potential publication or selective reporting bias was done using funnel plots, overall and by species category.

To investigate the factors associated with variation in seroprevalence, i.e. heterogeneity, meta-regression was done using a zero-inflated negative binomial model. The number of animals tested was used as the exposure variable, thus adjusting for differences in sample size and also appropriately weighting each group in the meta-regression. The negative binomial component modelled the number of positive reactors (i.e. the seroprevalence within an infected population), while the inflation component accounted for the excess number of zero outcomes by modelling the odds of the outcome being zero (i.e. the population not being infected). Predictor variables assessed were species, year, annual rainfall, serological test used and the degree of contact with livestock. The model was developed by backward elimination, with variables retained if significant (*P* < 0.05) or if they acted as confounders. Due to the likely differing nature of the wildlife-livestock interface for different wildlife species categories, the interaction term between species and livestock contact was also assessed. Robust (Huber-Eicker-White-sandwich) variances were used to account for clustering. The suitability of the negative binomial model compared to a Poisson model was assessed using a likelihood ratio test of the null hypothesis that the overdispersion parameter (*α*) equals zero. The fit of the zero-inflated negative binomial model compared to the regular negative binomial model was assessed using Akaike’s information criterion (AIC), with lower values indicating better fit. Meta-regression analysis was done using Stata 15.1 (StataCorp, College Station, TX, U.S.A.) and significance was assessed at *P* < 0.05.

## Supplementary Information

Supplementary Information 1.

Supplementary Information 2.
